# Supplementary material for: Predictive Factors for Difficult Laparoscopic Cholecystectomies in Acute Cholecystitis
Source: Diagnostics (Basel). 2024 Feb 5;14(3):346. doi: 10.3390/diagnostics14030346 (PMC10855974; doi:10.3390/diagnostics14030346)
Supplement: Supplementary file 1 [file diagnostics-14-00346-s001.zip › diagnostics-2843217-supplementary.pdf]

Table 1. Clinical and biological data of the patients included in the study groups:

Variable	Normal LC group (N = 150)	Difficult LC group (N = 105)	*p*-Value
**Age (mean±SD, years)**	52.4 (± 16.2)<br>Range: (19.0; 90.0)<br>52(IQR 12)	57.1 (± 14.2)<br>Range: (19.0; 83.0)<br>57(IQR 16)	0.009[a)]*
**Leukocytes (cells/microliter)**<br>(mean, range, median)	9622.5 (± 4170.5)<br>Range: (2100.0; 31000.0)<br>**8700.0 (IQR 4987.5)**	12731.6 (± 7216.8)<br>Range: (3900.0; 51200.0)<br>**11400.0 (IQR 7100.0)**	<0.001[a)]*
**Neutrophils (%)**<br>(mean, range, median)	0.7 (± 0.1)<br>Range: (0.3; 0.9)<br>**0.7 (IQR 0.1)**	0.7 (± 0.1)<br>Range: (0.1; 0.9)<br>**0.7 (IQR 0.3)**	0.001[a)]*
**Lymphocytes (%)**<br>(mean, range, median)	0.2 (± 0.1)<br>Range: (0.01; 0.6)<br>0.18 (IQR 0.17)	0.1 (± 0.1)<br>**Range: (0.004; 0.8)**<br>**0.13 (IQR 0.15)**	0.002 [a)]*
**NLR**<br>(mean, range, median)	6.3 (± 7.8)<br>Range: (0.4; 50.74)<br>**3.98 (IQR 4.8)**	11.06 (± 25.05)<br>Range: (0.02; 232.5)<br>**6.2 (IQR 6.72)**	0.001[a)]*
**Thrombocytes (cells/microliter)**<br>(mean, range, median)	247622.7 (± 81254.3)<br>Range: (74000.0; 763000.0)<br>**244500.0 (IQR 87000.0)**	264731.4 (± 174673.6)<br>Range: (68000.0; 1700000.0)<br>**240000.0 (IQR 103000.0)**	0.839 [a)]
**Fibrinogen (mg/dL)**<br>(mean, range, median)	409.8 (± 127.3)<br>Range: (189.4; 990.0)<br>**374.5 (IQR 129.03)**	574.5 (± 238.7)<br>Range: (206.0; 1220.0)<br>**570.0 (IQR 351.0)**	<0.001 [a)]*
**INR (UI/L)**<br>(mean, range, median)	1.2 (± 0.3)<br>Range: (0.9; 3.7)<br>**1.15 (IQR 0.16)**	1.3 (± 1.2)<br>Range: (0.9; 13.2)<br>**1.21 (IQR 0.22)**	0.021 [a)]*

**AST (UI/L)** (mean, range, median)	54.6 (± 86.4)<br>Range: (2.4; 664.0)<br>**28.0 (IQR 28.75)**	57.5 (± 99.9)<br>Range: (7.0; 952.0)<br>**30.0 (IQR 44.0)**	0.532 [a)]
**ALT (UI/L)** (mean, range, median)	74.8 (± 90.6)<br>Range: (1.3; 532.0)<br>**46.0 (IQR 44.0)**	70.8 (± 89. 4)<br>Range: (11.0; 627.0)<br>**37.0 (IQR 53.0)**	0.17 [a)]
**Total Bilirubin (mg/dL)** (mean, range, median)	1.1 (± 1.3)<br>Range: (0.2; 10.4)<br>**0.7 (IQR 0.6)**	1.2 (± 1.9)<br>Range: (0.2; 11.1)<br>**0.7 (IQR 0.6)**	0.715 [a)]
**Creatinine (mg/dL)** (mean, range, median)	0.8 (± 0.3)<br>Range: (0.3; 3.5)<br>**0.8 (IQR 0.2)**	0.9 (± 0.5)<br>Range: (0.4; 3.1)<br>**0.82 (IQR 0.3)**	0.187 [a)]
**Tongyoo score**	2.9±1.4<br>Range: (0; 6.0)<br>2.5(IQR 2.0)	6.0(± 2.6)<br>Range: (2.0; 13.0)<br>6.0(IQR 2.1)	<0.001[a)] *

Footnote: [a)] p- value was assessed with the Mann-Whitney. The alpha risk was set to 5% (α = 0.05); [b)] p-value was tested with the Chi-squared test. The alpha risk was set to 0.05; *statistically significant; ASA - The American Society of Anesthesiologists (ASA) physical status classification system; NLR: neutrophil-to-lymphocyte ratio; CBD: common bile duct.

Table 2. Imagistic data of the patients included in the study

Variable	Normal LC (N = 150)	Difficult LC (N = 105)	*p*-Value
CBD (mm)<br>(mean, range, median)	5.0 (± 2.3)<br>Range: (3.0; 18.0)<br>4.0 (IQR 1.0)	5.0 (± 2.0)<br>Range: (3.0; 13.0)<br>4.0 (IQR 1.0)	0.546[a)]
Transverse diameter of GB (mm) (mean, range, median)	28.8 (± 7.9)<br>Range: (3.0; 58.0)<br>29.5 (IQR 7.0)	36.5 (± 12.3)<br>Range: (15.0; 77.0)<br>35.0 (IQR 12.0)	<0.001[a)*]
Length of GB (mm)<br>(mean, range, median)	79.5 (± 18.6)<br>Range: (25.3; 140.5)	93.3 (± 27.2)<br>Range: (40.1; 175.4)	<0.001[a)*]

	79.7 (IQR 24.7)	90.3 (IQR 40.0)	
Thickness of GB wall (mm) (mean, range, median)	4.2 (± 1.5)<br>Range: (2.0; 10.0)<br>4.0 (IQR 2.0)	6.1 (± 2.9)<br>Range: (2.0; 22.0)<br>6.0 (IQR 3.0)	<0.001[a)*]

Footnote: [a)] p- value was assessed with the Mann-Whitney. The alpha risk was set to 5% (α = 0.05); [b)] p-value was tested with the Chi-squared test. The alpha risk was set to 0.05; *statistically significant;
